# Portal-mesenteric vein resection for pancreatic cancer: Results in par with the defined benchmark outcomes

**DOI:** 10.3389/fsurg.2022.1069802

**Published:** 2023-01-10

**Authors:** Gregory G. Tsiotos, Nikiforos Ballian, Fotios Milas, Panoraia Ziogou, Dimitrios Papaioannou, Charitini Salla, Ilias Athanasiadis, Flora Stavridi, Alexios Strimpakos, Maria Psomas, Georgia Kostopanagiotou

**Affiliations:** ^1^ Departments of Surgery, Mitera-Hygeia Hospitals, Athens, Greece; ^2^ Departments of Pathology, Mitera-Hygeia Hospitals, Athens, Greece; ^3^ Departments of Cytology, Mitera-Hygeia Hospitals, Athens, Greece; ^4^ Departments of Medical Oncology, Mitera-Hygeia Hospitals, Athens, Greece; ^5^ Departments of Anesthesiology, Mitera-Hygeia Hospitals, Athens, Greece

**Keywords:** borderline pancreatic cancer, portal vein resection, mesenteric vein resection, locally advanced pancreatic cancer, pancreaticoduodenectomy, benchmark outcomes

## Abstract

**Background:**

Patients with pancreatic cancer (PC), which may involve major peripancreatic vessels, have been generally excluded from surgery, as resection was deemed futile. The purpose of this study was to analyze the results of portomesenteric vein resection in borderline resectable or locally advanced PC. This study comprises the largest series of such patients in Greece.

**Materials and Methods:**

Investigator-initiated, retrospective, noncomparative study of patients with borderline resectable or locally advanced adenocarcinoma undergoing pancreatectomy en-block with portal and/or superior mesenteric vein resection in a tertiary referral center in Greece between January 2014 and October 2021. Follow-up was complete up to December 2021. Operative and outcome measures were determined.

**Results:**

Forty patients were included. Neoadjuvant therapy was administered to only 58% and was associated with smaller tumor size (median: 2.9 cm vs. 4.2 cm, *p* = 0.004), but not with increased survival. Though venous wall infiltration was present in 55%, it was not associated with tumor size, or Eastern Cooperative Oncology Group (ECOG) status. Resection was extensive: a median of 27 LNs were retrieved, R0 resection rate (≥1 mm) was 87%, and median length of resected vein segments was 3 cm, requiring interposition grafts in 40% (polytetrafluoroethylene). Median ICU stay was 0 days and length of hospitalization 9 days. Postoperative mortality was 2.5%. Median follow-up was 46 months and median overall survival (OS) was 24 months. Two-, 3- and 5-year OS rates were 49%, 33%, and 22% respectively. All outcomes exceeded benchmark cutoffs. Lower ECOG status was positively correlated with longer survival (ECOG-0: 32 months, ECOG-1: 24 months, ECOG-2: 12 months, *p* = 0.02).

**Conclusion:**

This series of portomesenteric resection in borderline resectable or locally advanced PC demonstrated a median survival of 2 years, extending to 32 months in patients with good performance status, which meet or exceed current outcome benchmarks.

## Introduction

Novel neoadjuvant therapies (NAT) have radically changed the approach to locally advanced and borderline resectable (LA, BR) (1) pancreatic adenocarcinoma (PC) leading to improved tumor responses, downstaging, and significant chances of curative resection. In conjunction with more aggressive surgery, including vascular resections, improved outcomes have been achieved (2–8).

However, this reality has not yet changed oncology practice in Greece. Pessimism prevails in the Greek oncology community, so that patients with non-metastatic BR/LA tumors are still considered to have a definitively “unresectable” tumor. These patients are managed with palliative intent and not referred to an advanced pancreatic center. In this complex context, our team, dedicated to advanced pancreatic surgery, adopted a multidisciplinary approach to PC as a tertiary referral center. Major vascular resections began in 2012. Our preliminary experience with unselected patients and scarce NAT administration indicated that patients with advanced Eastern Cooperative Oncology Group performance status (ECOG-PS) had dismal postoperative survival, whereas those with good ECOG-PS exceeded 2.5 years. With improved patient selection, NAT became more frequent and our technique was standardized. The aim of this study is to analyze our surgical, oncological, and long-term outcomes of these patients.

## Material and methods

Data on all patients who underwent pancreatectomy with resection of a portion of the superior mesenteric (SMV) and/or portal vein (PV) for tumor involvement at our division (>30 pancreatectomies/year) between 1/2014–10/2021 were prospectively collected and retrospectively analyzed. Only patients with PC were included. Clinicopathologic data, perioperative course, and complication data were recorded. Follow-up was complete to December 2021. The study was approved by our Institutional Review Board.

**Figure 1:**
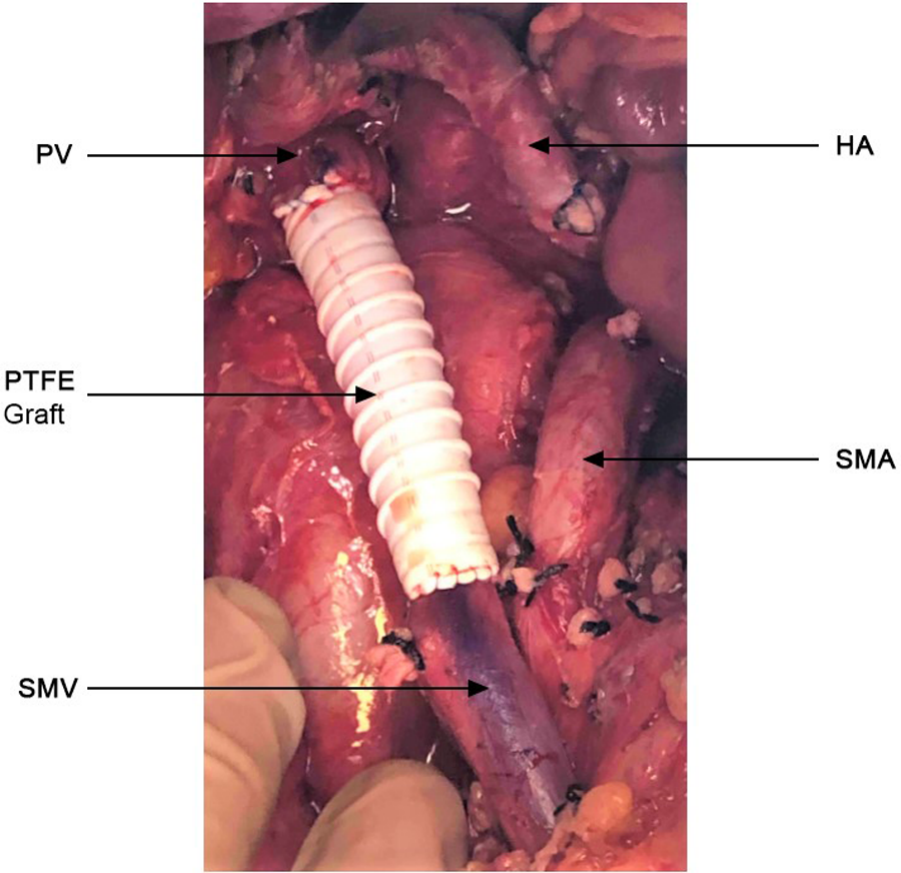
Complete skeletonization of the superior mesenteric vein (SMV) from its first tributaries deep within the mesentery, portal vein (PV) up to the liver hilum, hepatic artery (HA) and superior mesenteric artery from their take off. PTFE graft (4.5 cm) placed at resected portion of the SMV-PV.

All patients were staged preoperatively with pancreatic protocol computerized tomography with 2 mm sections. A PC was deemed BR or LA per the NCCN criteria (1). Intraoperatively, we completely skeletonized the PV, SMV and hepatic artery (Figure 1), as well as the celiac artery in these patients with pancreatic body tumors. Lymph node (LN) dissection included all standard peripancreatic LN beds. When venous involvement was minimal, a tangential longitudinal vein excision was performed and repaired transversely. When a circumferential vein segment was resected, the Cattell-Braasch maneuver (right-sided medial visceral rotation) was performed to approximate proximal and distal vein segments. This allowed a primary end-to-end anastomosis for venous gaps <3 cm, whereas interposition prosthetic polytetrafluoroethylene (PTFE) grafting was necessary for gaps >3 cm. We preferred prosthetic over autologous venous interposition grafts, as these were readily available, avoiding additional operative time for native vein harvesting. Their safety and long-term patency in this setting has been extensively demonstrated (9–11). In patients with tumor involvement of the splenomesenteric venous confluence, the splenic vein was not reimplanted and total pancreatectomy with splenectomy was performed. Daily aspirin was prescribed in these patients for life. With locally extensive disease, we proceeded to total pancreatectomy (TP) when appropriate. Patency of all prosthetic grafts was examined with ultrasonography 2 months postoperatively.

Histopathologic assessment of pancreatectomy specimens was performed, using standard guidelines of the College of American Pathologists, with R0 resection defined as negative margins >1 mm (https://documents.cap.org/protocols/cp-pancreas-exocrine-17protocol-4001.pdf).

Descriptive methods were used for continuous data given as median and interquartile range (IQR). Tests to assess normal distribution for numerical data were applied. Comparison of continuous variables between groups was performed using the Wilcoxon Mann-Whitney *U*-test. Categorical data are expressed as frequencies and percentages. Comparison of categorical variables among groups was performed predominantly using Fisher's Exact test. Follow-up duration was calculated with the reverse Kaplan-Meier method. Survival was calculated from the time of diagnosis for all patients (with or without NAT) to the time of death, or last follow-up. Survival curves were plotted using the Kaplan-Meier method. Differences in OS between groups were analyzed by the log-rank test. Multivariate survival analyses were also performed using the backward conditional Cox regression method; with proportionality verified by graphical assessment of Kaplan-Meier curves. *p* < 0.05 was considered statistically significant. All tests used were two-tailed. Statistical analysis was performed with the SAS statistical software vs. 7.1.

## Results

### Demographic and perioperative data

During the study period, forty patients (29 with BR and 11 with LA) underwent pancreatectomy with resection of some part of the SMV and/or PV. Clinicopathologic characteristics are shown in Table 1. TP, the most common resection (55%), was performed in all 12 patients with neck tumors, in 2 with large body tumors which extended to the neck, and in 4 with large head tumors involving and extending beyond the neck. Four additional patients with uncinate tumors underwent TP because these extended anteriorly towards the neck, completely involving the splenomesenteric venous junction. Eight patients with uncinate tumors and 4 with head tumors underwent a Whipple operation (30%). The remaining 6 with body tumors underwent a distal pancreatectomy (15%). After venous resection, the SMV-PV was reconstructed with a transverse lateral venorrhaphy in 8 patients (20%), with a primary end-to-end anastomosis (16 patients, 40%), or with an interposition PTFE graft (16 patients, 40%) (Table 1).

**Table 1:** Clinicopathologic characteristics of 40 patients who underwent pancreatectomy with PV/SMV resection for BR/LA PC. ICU: intensive care unit, LOS: length of stay, LN: lymph node.

		*N* (%)
Gender	Female	18 (45)
	Male	22 (55)
**Age, years [median (IQR)]**		65 (59–71)
ECOG	0	16 (40)
	1	14 (35)
	2	10 (25)
Location	Body	8 (20)
	Head	8 (20)
	Neck	12 (40)
	Uncinate	12 (40)
Neoadjuvant Chemotherapy	No	17 (42)
	Yes	23 (58)
Operation	Distal	6 (15)
	Total	22 (55)
	Whipple	12 (30)
Venous Reconstruction Type	Primary	16 (40)
	PTFE	16 (40)
	Lateral venorrhaphy	8 (20)
**Length resected, cm [median (IQR)]**		3 (2–4)
ICU stay	No	24 (83)
	Yes	7 (18)
**ICU stay, days [median (IQR)]**		0 (0–0)
**Transfused pRBC units [median (IQR)]**		2 (0–3)
**Postoperative hospital LOS, days [median (IQR)]**		9 (7–14)
Adjuvant Chemotherapy	No	22 (62)
	Yes	15 (38)
**Tumor size, cm [median (IQR)]**		3.2 (2.5–4.1)
T	T1	7 (18)
	T2	14 (35)
	T3	19 (48)
N	N0	6 (15)
	N1	28 (70)
	N2	6 (15)
Resection	R0	35 (87)
	R1	5 (13)
	R2	0 (0)
**Total LNs [median (IQR)]**		27 (17–35)
**Positive LNs [median (IQR)]**		2 (1–5)
**LN ratio, % [median (IQR)]**		10 (0–20)
Vein infiltration	No	18 (45)
	Yes	22 (55)
Complications	No	28 (70)
	Yes	12 (30)
Reoperation	No	35 (87)
	Yes	5 (13)

The duration of operation in our hospital is recorded not as “skin-to-skin” time, but from the time of patients' entry to until exit from the operating room. Thus, median OR time was 560 min (IQR: 470–635), or 9.3 h (IQR: 7.8–10.6). Twenty-seven patients (68%) received at least one unit of PRBCs, for a median of 2 (IQR: 0–3).

Adjuvant chemotherapy (Gemcitabine alone) was administered to 15/39 (38%) discharged patients. The remaining 24 (62%) did not receive chemotherapy because they had undergone NAT (*n* = 22), or were unfit (*n* = 2).

### Neoadjuvant chemotherapy

No patient received chemoradiation. Twenty-three patients (58%) received NAT: 11 Gemcitabine/nab-paclitaxel, 11 FOLFIRINOX, and 1 both regimens. Eight (35%) were treated elsewhere (4 Gemcitabine/nab-paclitaxel, 3 FOLFIRINOX, and 1 both) and NAT details could not be confirmed. The remaining 15 (65%) were treated at our institution (7 Gemcitabine/nab-paclitaxel, 8 FOLFIRINOX) for a median of 6 cycles (range: 2–12, IQR: 4–6). The decision to operate on patients receiving NAT was based on completion of the 6-month protocol, or on their inability to continue until completion, provided (in all) that there was associated decrease of CA 19–9 and no disease progression by CT criteria.

Seventeen patients did not undergo NAT, or full-term NAT. Some deemed resectable upfront, revealed venous invasion intraoperatively. Other self-referred patients had undergone heterogeneous regimens of inappropriate (choice of chemotherapeutic agents, reduced doses, frequency, or number of cycles), palliative chemotherapy elsewhere. These patients either declined appropriate NAT, or were re-evaluated to have resectable disease by CT and CA 19–9 criteria. We classified all these patients as pancreatectomy without prior NAT.

The administration of NAT was associated with smaller tumor size (median: 2.9 cm vs. 4.2 cm, *p* = 0.04), and less associated with vein wall infiltration (35% vs. 65%, *p* = 0.06), but did not correlate with number of resected or positive LNs, LN ratio, type or length of vascular resection, ECOG status, or survival (NAT vs. no NAT, 23 vs. 25 months, *p* = 0.7).

### Pathologic findings

Median tumor size was 3.2 cm (IQR: 2.5–4.1). Sixteen patients had tumors <3 cm and 11 (69%) of those had received NAT. The median number of LNs harvested was 27 (IQR: 17–35). A median of 2 LNs (IQR: 1–5) was positive, for a median LN ratio of 10% (IQR: 0%–20%). The resected veins were histologically infiltrated in most patients (22, 55%), whereas in the remaining 18 (45%) the vessel wall was densely adhered to, but not infiltrated by cancer. Of note, 14 of the latter 18 patients (78%) had undergone NAT. Indeed, vein wall infiltration was less frequent among patients after NAT (35% vs. 82%, *p* = 0.06), but showed no correlation with ECOG status, or tumor size. R0 resection was achieved in 35 patients (87%) and R1 in 5 (13%). Portomesenteric vein margin was positive (<1 mm) in 2 of the patients with R1 resection (5% of all patients).

### Morbidity and mortality

Prosthetic graft patency on 2-month postoperative ultrasound was 100%. One of the 16 patients (6%) with PTFE graft, developed upper gastrointestinal bleeding 19 months postoperatively requiring admission and blood transfusion. CT scan demonstrated graft occlusion, dilated mesenteric veins and collaterals in the liver hilum. She had no recurrent hemorrhage, but died 5 months later due to disease progression. In the remaining 15 patients with PTFE grafts, no signs of infection, thrombosis, or anastomotic breakdown were encountered. Twelve patients (30%) developed at least one major (Clavien-Dindo grade ≥ 3A) complication: hemorrhage (1, or 2.5%), wound dehiscence (3), grade B pancreatic fistula (2 of 18 patients with Whipple or DP, 11%), bile leak (3), transverse colon necrosis (1), gastric staple line leak (1), delayed gastric emptying (2), and hepatic artery spasm with intrahepatic cholestasis and liver failure (1).

Five patients (13%) required reoperation and one (2.5%) a major intervention. Those with postoperative hemorrhage, wound dehiscence, gastric leak, and colon necrosis were reoperated and did well. The patient with hepatic artery spasm was subjected to emergent hepatic artery stenting, but died 30 days postoperatively (mortality 2.5%) because of rapidly progressing intrahepatic cholestasis and liver failure. Fourteen of the 39 discharged patients (36%) required hospital readmission within a year from operation.

### Survival

**Figure 2:**
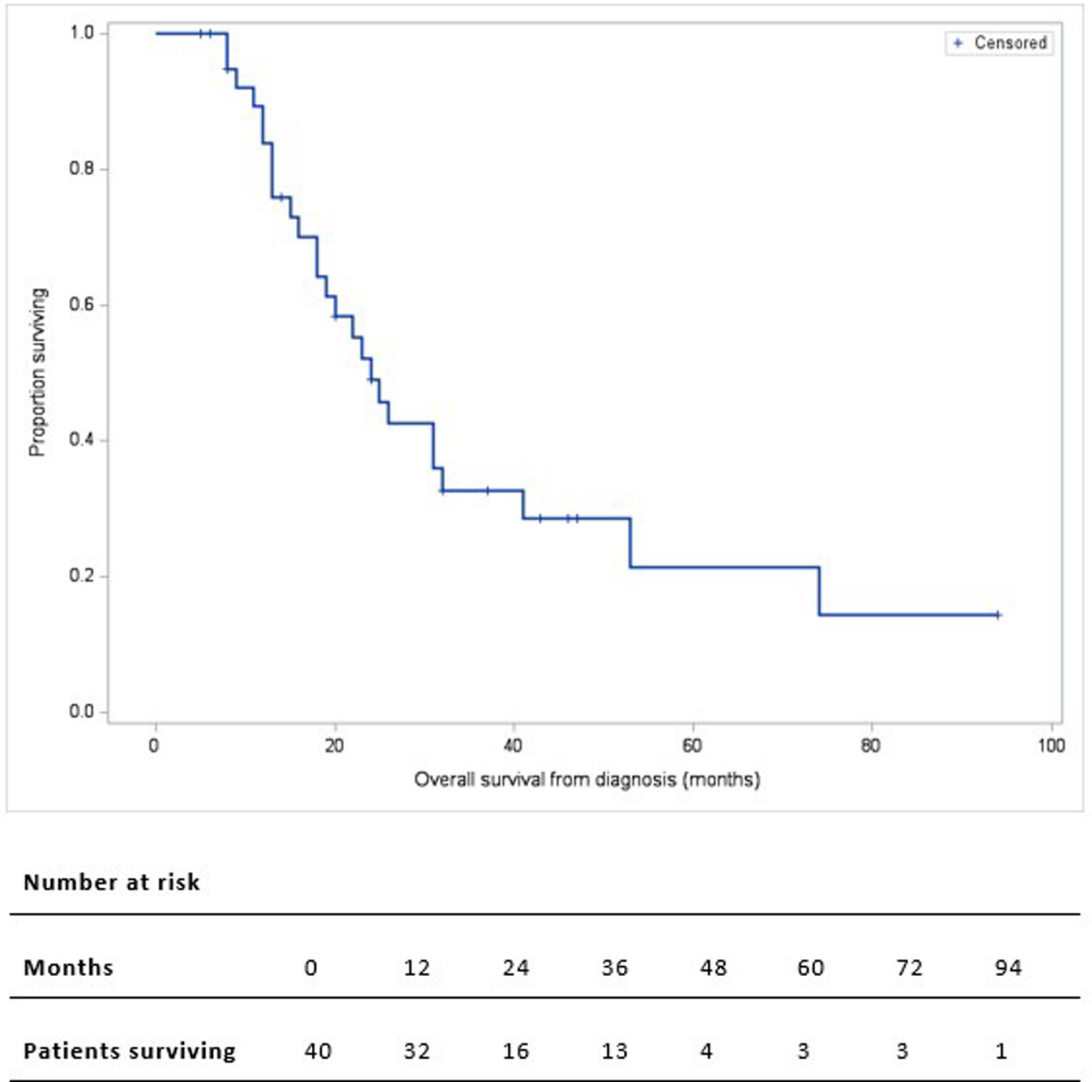
Kaplan-Meier overall survival curve of 40 patients who underwent pancreatectomy with PV/SMV resection for BR/LA PC. Overall survival is calculated from the time of diagnosis.

**Figure 3:**
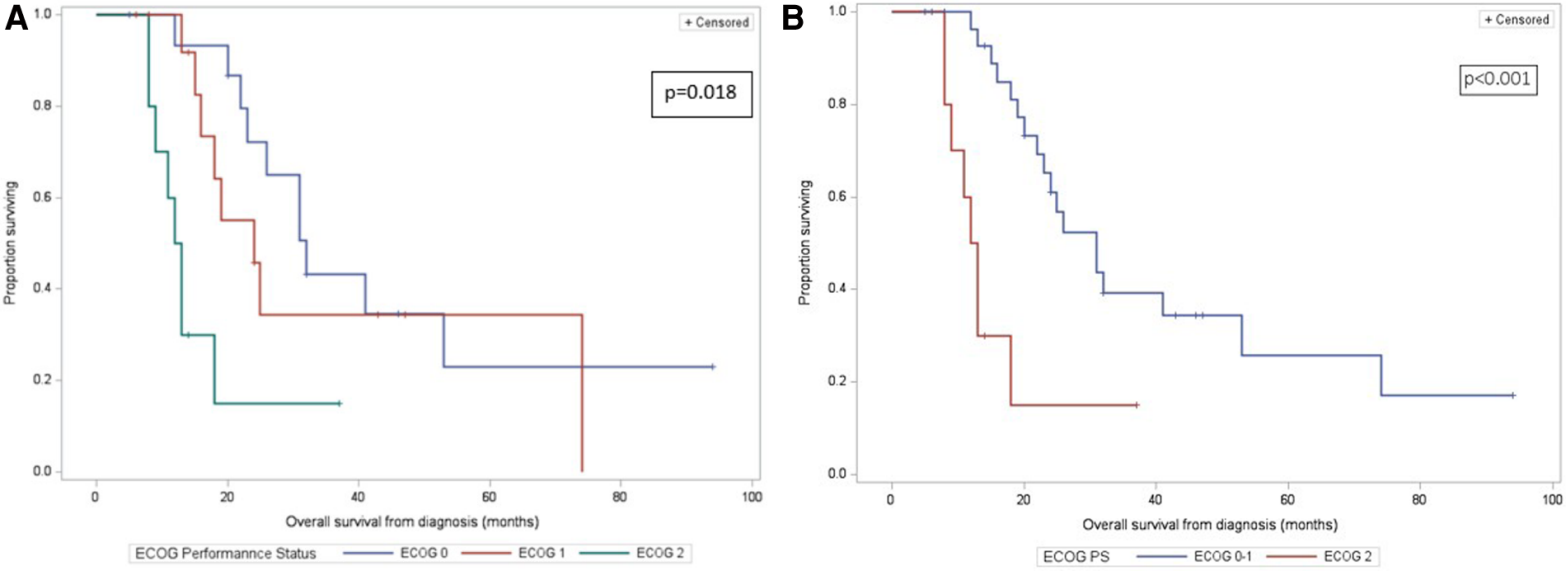
(**A**) kaplan-meier overall survival curves of 40 patients who underwent pancreatectomy with PV/SMV resection for BR/LA PC, by ECOG-0/1/2 category. Overall survival is calculated from the time of diagnosis. (**B**) Kaplan-Meier overall survival curves of 40 patients who underwent pancreatectomy with PV/SMV resection for BR/LA PC, by ECOG-0 or −1 vs. ECOG-2 category. Overall survival is calculated from the time of diagnosis.

The median follow-up duration was 46 months (IQR: 32–94). Of the 39 discharged patients, one died of COVID-19 complications (being free of PC) and 24 died of metastatic disease: liver metastases (20, 80%), or peritoneal carcinomatosis (4, 20%). Two-, 3-, and 5-year OS rate was 49%, 33%, and 22% respectively. The median OS from the time of diagnosis was 24 months (Figure 2). Higher ECOG status was significantly associated with shorter survival on univariate analysis (median OS from time of diagnosis [IQR]: ECOG-0: 32 months [25–53], ECOG-1: 24 months [16–74], ECOG-2: 12 months [9–18], *p* = 0.018) (Figure 3A). Patients with ECOG-0 and −1, grouped together, also had significantly better survival than those with ECOG-2: 31 months [20–74] vs. 12 months [9–18], *p* < 0.01. (Figure 3B).

## Discussion

NAT and PV/SMV resection have been important advances in the treatment of BR/LA PC. Following NAT, 20%–60% of patients with LA disease undergo resection leading to prolonged survival (2, 4, 5, 12, 13), thus this scheme is the guideline of the International Study Group of Pancreatic Surgery (ISGPS) (14). Recently, more radical surgery including PV/SMV resection is increasingly performed (3, 5–8, 15, 16); it has contributed to improved outcomes with acceptable mortality (3%–5%) in referral centers with survival comparable to that of pancreatectomy with no vein involvement (3, 4, 6, 7). In fact, benchmark outcomes for pancreatoduodenectomy with portomesenteric vein resection were recently established by an international group of experts (17).

Despite this reality, pessimism still exists in Greece: most patients with BR/LA PC are channeled to palliative chemotherapy as the sole treatment. Such was the context in which we began performing vascular resections in 2012. Our initial efforts led to promising outcomes, despite inclusion of all patients. In an unselected cohort with limited NAT administration, patients with ECOG-0 had a median survival of 33 months. Following standardization of patient selection and technique, herein we report on our 40 most recent BR/LA PC patients undergoing major vein resection from 2014 to 2021.

In this group, increasing acceptance of NAT resulted in smaller tumor size and less frequent vein wall infiltration. Extensive oncologic resections were performed with 27 LNs retrieved, 87% R0 rate, and long vein segments resected (3 cm) with almost half of them requiring interposition grafts. PTFE grafts did not significantly increased long-term morbidity. Postoperative mortality was 2.5% and median OS was 24 months. These results appear promising despite the lack of universal and uniform NAT. Although our study included not only Whipple procedures, but also total pancreatectomies (for very extensive tumors) and a few distal pancreatectomies, it is worth noting that our outcomes meet (or exceed) the benchmark cutoffs recently established for pancreatoduodenectomy with PV/SMV resection (17). This is true for most of the benchmarked variables including hospital and ICU stay, major postoperative complications, mortality, PV occlusion, and survival (Table 2).

**Table 2:** Comparison of our results with the benchmark outcome for pancreatoduodenectomy and PV/SMV resection. (Our “Operation duration” includes the total time of patients’ presence in the OR; not “skin-to-skin” time, n/a: not assessed).

	Benchmark cutoffs	Our experience
Operation duration	≤8 h	(9.3 h)
Intraop. Blood transfusion rate	≤27%	68%
ICU stay	≤1 day	0 day
Hospital stay	≤14 day	9 day
**Complications**		
Clavien-dindo grade ≥3A	≤28%	30%
POPF-B/C	≤14%	11%
Postop. Bleeding grade ≥3	≤7%	2.5%
In-hospital mortality	≤4%	2.5%
1-year hospital readmission rate	≤32%	36%
Portal vein occlusion	≤4%	2.6%
**Resection margin**		
R0	≥35%	87%
R1	≤63%	13%
R2	≤2%	0
(+) PV margin	≤3%	5%
Total number of lns resected	≥16	27
**Overall survival rate**		
1-year	≥68%	n/a
2-year	≥37%	49%
3-year	≥21%	33%
5-year	≥9%	22%

The extent of LN dissection has been standardized for a Whipple operation (18, 19). Fifteen LNs are considered oncologically adequate, with 20 LNs recommended in chemotherapy-naïve patients (16, 19). In our specimens, a median of 27 LNs were retrieved, securing extensive peripancreatic tissue clearance and comparing favorably to most pancreatic centers (17).

The extent of our resections is reflected also in the 87% R0 resection rate, similar to the 55%–96% “negative microscopic margin” rate reported by others (2, 20–22), when considering that in those studies all patients had undergone NAT (versus only 58% in ours) and margins <1 mm were considered negative (R0). A little over half of our patients had to undergo TP given their tumor extent and the absence of “downstaging” in many. In this era of more extensive pancreatic surgery, TP has indeed become more frequent (23).

The lack of NAT in many of our patients and the larger tumor size thereof was probably associated with the longer segments of PV/SMV resected. Thus, 40% of our patients needed an interposition graft. In contrast, in the Mayo Clinic (24) and Heidelberg (16) experience, only 16% and 18% respectively had removed vein segments long enough to necessitate a graft. Others have reported interposition grafts in 33%–45% of patients (9, 25). The safety, efficacy and long-term patency of PTFE grafts have been studied and documented (9–11). Our good experience with PTFE utilization in cases with long venous gaps certainly corroborates published data.

Vein wall infiltration was histologically present 55% of the time, similar to others' experience of 51%–93% (9, 15, 16, 26). In our group, NAT-naïve patients were more likely (*p* = 0.06) to have their resected vein histologically infiltrated (82% vs. 18%). Because of the notorious lack of correlation between radiographic, operative, and pathologic findings after NAT (12, 15, 21, 27), our strategy has been to proceed with attempt at resection based on the significant CA 19–9 decrease (20), even if the tumor is radiographically “stable”.

Median OS with upfront surgery without NAT ranged from 15 to 23 months (26, 28). Following NAT, it reached 2 years (22, 29). Consensus has been reached that NAT is an absolute prerequisite in BR/LA PC before resection is contemplated (14). In centers of excellence and highly selected patients, median survival may now exceed 3 years (4, 13, 21, 30). Our median OS of 24 months (32 months for ECOG-0) compares favorably with the literature, since only 58% of our group received NAT. The absence of universal and uniform NAT in our group may have contributed to its failure to significantly increase survival. ECOG ≥ 2 has been recognized as a negative prognostic factor after pancreatectomy (31). Indeed, this proved to be true in our group as patients with ECOG-2 had markedly shorter survival (12 months) compared to those with ECOG-0 (32 months), ECOG-1 (24 months), and the combination of ECOG-0 and −1 (31 months). Appropriate prehabilitation may upstage ECOG and contribute to longer postoperative survival (32).

Several weaknesses of our study should be acknowledged. Patient numbers and heterogeneity of prior NAT receipt did not allow meaningful comparison of survival between patients who did and did not undergo NAT (23 vs. 25 months, *p* = 0.7). It is our strong conviction that all patients with LA/BR pancreatic adenocarcinoma should undergo neoadjuvant chemotherapy before being considered for pancreatectomy. This has been extensively proven in prospective studies with significant patient samples. In addition, data on some benchmark criteria proposed by Raptis et al., were not being collected prospectively until publication of their manuscript and, hence, are absent from the present report.

## Conclusions

Our current experience with pancreatectomy and PV/SMV resection for BR/LA PC comprised a group of patients, many of whom did not receive NAT, who underwent extensive dissections, did not need ICU admission, required minimal blood transfusions, and had 2.5% mortality and a median OS of 24 months, reaching 32 months for ECOG-0. Our outcomes are in par with those reported from other centers, or exceed established outcome benchmarks. Although we certainly need to generalize NAT, improve patient selection and prehabilitate ECOG-1/2 patients, these results show that survival in patients with BR/LA PC can indeed be prolonged after appropriate extensive resections. These results should provoke more BR/LA PC patients to undergo modern neoadjuvant protocols with the goal of curative resection and further survival improvement.

## Data Availability

The raw data supporting the conclusions of this article will be made available by the authors, without undue reservation.
